# The impact of concomitant chronic total occlusion on clinical outcomes in patients undergoing transcatheter aortic valve replacement: a large single-center analysis

**DOI:** 10.3389/fcvm.2024.1338253

**Published:** 2024-02-23

**Authors:** Maximilian Will, Konstantin Schwarz, Thomas Weiss, Gregor Leibundgut, Elisabeth Schmidt, Paul Vock, Roya Mousavi, Josip A. Borovac, Chun Shing Kwok, Uta C. Hoppe, Julia Mascherbauer, Gudrun Lamm

**Affiliations:** ^1^ Karl Landsteiner University of Health Sciences, Krems, Austria; ^2^ Division of Internal Medicine 3, University Hospital St. Pölten, St. Pölten, Austria; ^3^ Karl Landsteiner Institute for Cardiometabolics, Karl Landsteiner Society, St Poelten, Austria; ^4^ Medical School, Sigmund-Freud University, Vienna, Austria; ^5^ Klinik für Kardiologie, Universitätsspital Basel, Basel, Switzerland; ^6^ Cardiovascular Diseases Department, University Hospital of Split, Split, Croatia; ^7^ Department of Post-Qualifying Healthcare Practice, School of Nursing and Midwifery, Birmingham City University, Birmingham, United Kingdom; ^8^ Department of Cardiology, University Hospitals of North Midlands NHS Trust, Stoke-on-Trent, United Kingdom; ^9^ University Department of Internal Medicine II, Cardiology and Internal Intensive Care Medicine, Paracelsus Medical University, Salzburg, Austria

**Keywords:** Coronary artery disease (CAD), aortic stenosis (AS), transcatheter aortic valve replacement (TAVR), chronic total occlusion (CTO), percutaneous coronary intervention (PCI)

## Abstract

**Background:**

Coronary artery disease (CAD) is a common finding in patients with severe aortic stenosis undergoing transcatheter aortic valve replacement (TAVR). However, the impact on prognosis of chronic total occlusions (CTOs), a drastic expression of CAD, remains unclear.

**Methods and results:**

We retrospectively reviewed 1,487 consecutive TAVR cases performed at a single tertiary care medical center. Pre-TAVR angiograms were analyzed for the presence of a CTO. At the time of TAVR, 11.2% (*n* = 167) patients had a CTO. There was no significant association between the presence of a CTO and in-hospital or 30-day mortality. There was also no difference in long-term survival. LV ejection fraction and mean aortic gradients were lower in the CTO group.

**Conclusions:**

Our analysis suggests that concomitant CTO lesions in patients undergoing TAVR differ in their risk profile and clinical findings to patients without CTO. CTO lesion *per se* were not associated with increased mortality, nevertheless CTOs which supply non-viable myocardium in TAVR population were associated with increased risk of death. Additional research is needed to evaluate the prognostic significance of CTO lesions in TAVR patients.

## Introduction

In the past few decades, transcatheter aortic valve replacement (TAVR) has become a cornerstone in the management of severe aortic stenosis (AS) (1–5). Historically, the use of TAVR began as a treatment of patients who were classified at a high or prohibitive risk for cardiac surgery. However, there is now evidence that TAVR may outperform surgical valve replacement even in low surgical risk patients (1) and TAVR procedures surpass surgical valve replacements in terms of annual frequency in the United States (6, 7).

Risk factors of degenerative calcific AS are comparable to those that contribute to coronary artery disease (CAD) (8, 9), leading to common coexistence of both conditions (10, 11).

Current guidelines (2, 12) suggest that coronary revascularization should be considered in patients with prognostically significant CAD prior to aortic valve intervention because untreated CAD may be linked to negative outcomes in patients receiving TAVR (13). However, beside the lack of high-quality evidence for the question if revascularization should be offered for patients with severe AS, optimal timing, extent, and modality of coronary revascularization remain unclear (2, 12, 14–17).

Chronic total occlusions (CTO) of coronary arteries represent a common finding in patients with chronic coronary syndromes (CCS) (18). There is an increasing amount of research suggesting that CTOs have adverse effects and increased mortality rates among various CAD populations (19–21). These findings may apply to patients undergoing TAVR due to several underlying mechanisms (22–24), but most randomized controlled trials (RCTs) and large registries investigating TAVR outcomes do not include detailed information on CTOs (25). As such, current evidence related to the prognostic significance of a CTO among TAVR patients is sparse.

To date, only a few available studies with conflicting results exist (26–28). A recent meta-analysis reported increased in-hospital complications in patient with concomitant CTO lesions undergoing TAVR (29). On the other hand, the presence of CTO by itself was not associated with increased long-term mortality (29).

Therefore, the principal aim of the current study was to examine potential differences in relevant characteristics among patients with severe AS that presented with or without CTO. Secondary aim was to evaluate the prognostic relevance of CTO lesion in TAVR patients in a large single center cohort.

## Methods

We retrospectively reviewed consecutive TAVR cases performed at a single tertiary care academic medical center from January 2016 until December 2021. We included cases with pre-TAVR coronary angiograms (CAG) that were accessible for review. The majority of patients underwent a diagnostic CAG weeks or months prior to the TAVR procedure. The decision whether to perform revascularization or to pursue conservative treatment was made by the treating physician. All revascularization procedures were performed prior TAVR. Each angiogram was reviewed by at least one interventional cardiologist (ES, MW, KS) for the presence or absence of CTO following current criteria. If a CTO lesion was present, detailed information of the coronary anatomy was described. Patients with normal ventricular function and no regional wall motion abnormalities as determined by echocardiography are considered to have intact myocardial viability. In instances where these conditions are not met, viability testing is conducted using cardiac MRI or PET imaging.

The primary endpoint was overall survival (reported as survival at median follow-up). Secondary endpoints included were in-hospital mortality, 30-day mortality (post-discharge), post-TAVR left ventricular (LV) function, and periprocedural complications.

Follow-up data for mortality rates were 99% complete, based on the local database data from Sankt Pölten University Hospital. Follow-up period began on the date of the TAVR procedure and concluded at the first occurrence of either death, emigration, or end of the study (31 December 2021).

Systolic function was assessed by left ventricular ejection fraction (LVEF) measured by the Simpson's biplane method from transthoracic echocardiography.

Periprocedural adverse events included acute renal failure with need for post-operative dialysis, post-operative stroke or transient ischemic attack, third-degree atrioventricular block or pacemaker requirement, aortic dissection, tamponade, circulatory arrest, gastrointestinal complications, a requirement for surgical intervention, periinterventional valvular dysfunction, annulus rupture, vascular complications, and, in extreme cases, operative mortality occurring during the surgical procedure. These parameters were evaluated according to Valve Academic Research Consortium-2 guideline recommendations (14). All information was abstracted from the electronic health records and stored on an institutional database. Mortality data were obtained from medical records as well as public death records. Comorbidities were based on common definitions derived from review of hospital records. Cardiac surgery risk prediction scores were calculated for each patient, and this included the calculation of EuroSCORE I model (additive and logistic) and EuroSCORE II model.

In accordance with standard definitions of significant flow-limiting stenoses, obstructive CAD was characterized as any stenosis 50% or greater in the left main, 70% or greater in any other coronary artery, or both (14–16). The extent of the CAD was classified as one-, two-, or three-vessel disease. CTO lesions were defined as occluded arteries with TIMI 0 antegrade flow and a clinically suspected or documented duration of at least 3 months.

### Statistical analysis

Statistical analysis was performed on STATA 13.0 (College Station, TX). The cohort of patients with severe AS was stratified by whether the patients had CTO or no CTO. Descriptive statistics were presented for demographic variables, comorbidities, clinical characteristics at index hospitalization, extent of CAD, creatinine, hemoglobin, surgical risk scores, LVEF, mean aortic valve gradient, aortic valve area as well as periprocedural variables and outcomes. Median and interquartile range were presented for continuous variables and number and percentages were used to describe categorical data. The Mann–Whitney *U*-test and *χ*^2^ test were used to determine potential statistical differences between CTO groups for continuous and categorical variables, respectively. Among patients with CTO further descriptive data were presented for whether patients received coronary revascularization, the vessel that was affected, the severity of CAD, and whether the myocardium was viable in the territory of the vessel. Additional subgroup analyses were performed to explore the effect of whether there was CTO or no CTO, revascularization, or no revascularization of the CTO, whether there was viability in the territory of CTO and whether there was revascularization of a CTO of the proximal LAD on post-operative outcomes.

The Institutional Ethics Review Board of Lower Austria approved this study (ethics commission number GS4-EK4/843-2023). The reporting of this study is in accordance with the STrengthening the Reporting of OBservational studies in Epidemiology (STROBE) recommendations (30).

## Results

Table 1 summarizes the characteristics and comorbidities of patients who underwent transcatheter aortic valve replacement (TAVR) with and without chronic total occlusion (CTO). A total of 1,320 patients without CTO and 167 patients with CTO were included in the analysis.

**Table 1:** Characteristic and comorbidities of patients with patient who undergo TAVR with chronic total occlusion vs. no chronic total occlusion.

Variable	No CTO (*n* = 1,320)	CTO (*n* = 167)	*p*-value
Median age (IQR)	81 (78–85)	81 (77–91)	0.94
Male	637 (48.3%)	129 (77.3%)	<0.001
Hypertension	1,040 (78.8%)	143 (85.6%)	0.039
Dyslipidaemia	869 (65.8%)	147 (88.0%)	<0.001
Diabetes mellitus	432 (32.7%)	67 (40.1%)	0.057
Angina	45 (3.4%)	14 (8.4%)	0.002
Acute myocardial infarction	30 (2.3%)	18 (10.8%)	<0.001
Percutaneous coronary intervention	470 (35.6%)	80 (47.9%)	0.002
Coronary artery bypass graft	24 (1.8%)	110 (65.9%)	<0.001
Previous valve intervention	52 (3.9%)	11 (6.6%)	0.11
Atrial fibrillation/flutter	494 (37.4%)	65 (38.9%)	0.71
Atrioventricular block	152 (11.5%)	27 (16.2%)	0.082
Left bundle branch block	112 (8.5%)	15 (9.0%)	0.83
Right bundle branch block	97 (7.4%)	8 (4.8%)	0.22
Stroke	138 (10.5%)	17 (10.2%)	0.91
Carotid artery disease	10 (0.8%)	6 (3.6%)	0.001
Previous carotid artery surgery	9 (0.7%)	7 (4.2%)	<0.001
Cerebrovascular disease	92 (7.0%)	27 (16.2%)	<0.001
Vascular surgery	24 (1.8%)	11 (6.6%)	<0.001
Renal disease	37 (2.8%)	10 (6.0%)	0.027
Dialysis	19 (1.4%)	2 (1.2%)	0.80
Chronic obstructive pulmonary disease	106 (8.0%)	10 (6.0%)	0.35
Malignancy	162 (15.6%)	14 (12.7%)	0.43
Neurological disease	48 (3.6%)	4 (2.4%)	0.41
New York Heart Association class			0.60
I	17 (1.3%)	2 (1.2%)	
II	50 (3.8%)	10 (6.0%)	
III	1,170 (88.6%)	145 (86.8%)	
IV	83 (6.3%)	10 (6.0%)	
Cardiogenic shock at presentation	11 (0.8%)	3 (1.8%)	0.23
Resuscitation	7 (0.5%)	1 (0.6%)	0.91
Coronary artery disease	578 (43.8%)	167 (100%)	<0.001
Left main stem disease >50%	54 (4.1%)	96 (57.5%)	<0.001
Vessel of coronary artery disease			<0.001
None	727 (55.1%)	0 (0%)	
Left anterior descending	308 (23.3%)	16 (9.6%)	
Left circumflex	161 (12.2%)	27 (16.2%)	
Right coronary	124 (9.4%)	124 (74.3%)	
Median creatinine (IQR)	1.1 (0.9–1.4)	1.2 (1.0–1.5)	0.007
Median haemoglobin (IQR)	12.7 (11.5–13.8)	12.8 (11.5–14.1)	0.56
Euroscore add	8 (7–9)	10 (9–12)	<0.001
Log Euroscore	10.1 (7.3–14.7)	18.1 (11.7–28.4)	<0.001
Euroscore 2	3.4 (2.0–4.9)	7.0 (4.2–11.5)	<0.001
Mean ejection fraction	53.8 ± 10.9	48.8 ± 12.8	<0.001
Mean AV mean gradient	45.8 ± 18.6	36.8 ± 14.8	<0.001
Aortic Valve Area (IQR)	0.7 (0.6–0.8)	0.7 (0.6–0.9)	0.052

The median age of the patients in both groups was similar, with the no CTO group having a median age of 81 years [interquartile range (IQR): 78–85], and the CTO group having a median age of 81 years (IQR: 77–91) (*p* = 0.94). Male gender was more prevalent in the CTO group, with 77.3% (*n* = 129) of patients being male, compared to 48.3% (*n* = 637) in the no CTO group (*p* < 0.001).

Regarding comorbidities, hypertension was present in a higher proportion of patients in the CTO group (85.6%, *n* = 143) compared to the no CTO group (78.8%, *n* = 1,040) (*p* = 0.039). Similarly, dyslipidemia was more prevalent in the CTO group (88.0%, *n* = 147) compared to the no CTO group (65.8%, *n* = 869) (*p* < 0.001). There was a trend towards a higher prevalence of diabetes mellitus in the CTO group (40.1%, *n* = 67) compared to the no CTO group (32.7%, *n* = 432), although it did not reach statistical significance (*p* = 0.057).

The presence of angina was significantly higher in the CTO group (8.4%, *n* = 14) compared to the no CTO group (3.4%, *n* = 45) (*p* = 0.002). Additionally, a higher proportion of patients in the CTO group had a history of acute myocardial infarction (10.8%, *n* = 18) compared to the no CTO group (2.3%, *n* = 30) (p < 0.001). Percutaneous coronary intervention was more frequently performed in the CTO group (47.9%, *n* = 80) compared to the no CTO group (35.6%, *n* = 470) (*p* = 0.002). Coronary artery bypass graft surgery was more prevalent in the CTO group (65.9%, *n* = 110) compared to the no CTO group (1.8%, *n* = 24) (*p* < 0.001).

The prevalence of other comorbidities, such as atrial fibrillation/flutter, atrioventricular block, left bundle branch block, right bundle branch block, stroke, carotid artery disease, previous carotid artery surgery, cerebrovascular disease, vascular surgery, renal disease, dialysis, chronic obstructive pulmonary disease, malignancy, and neurological disease, did not show significant differences between the two groups (*p* > 0.05 at all instances).

Notably, the CTO group had a higher proportion of patients with left main stem disease (>50%) compared to the no CTO group (57.5% vs. 4.1%, *p* < 0.001). In the no CTO group, 578 (43.8%) patients had CAD (p < 0.001). Furthermore, the distribution of the vessels involved in CAD differed significantly between the groups (*p* < 0.001).

Regarding laboratory parameters, the median creatinine levels were significantly higher in the CTO group (1.2 mg/dl, IQR: 1.0–1.5) compared to the no CTO group (1.1 mg/dl, IQR: 0.9–1.4) (*p* = 0.007). However, there was no significant difference in median hemoglobin levels between the two groups (*p* = 0.56).

The EuroSCORE model I (additive), EuroSCORE model I (logistic), and EuroSCORE II were all significantly higher in the CTO group compared to the no CTO group (*p* < 0.001 for all). The mean ejection fraction was significantly lower in the CTO group (48.8% ± 12.8%) compared to the no CTO group (53.8% ± 10.9%) (*p* < 0.001).

The mean aortic valve gradient was significantly higher in the no chronic total occlusion (CTO) group (45.8 ± 18.6 mmHg) compared to the CTO group (36.8 ± 14.8 mmHg) (*p* < 0.001). However, there was no statistically significant difference in the aortic valve area between the two groups, with a median value of 0.7 cm^2^ (IQR: 0.6–0.8) in the no CTO group and 0.7 cm^2^ (IQR: 0.6–0.9) in the CTO group (*p* = 0.052).

Table 2 presents the procedural variables and outcomes of patients who underwent TAVR with and without CTO. The median procedure time was similar in both groups, with a median of 27 min [interquartile range (IQR): 22–36] in the no CTO group an IQR: 22–37 in the CTO group (*p* = 0.83).

**Table 2:** Procedural variables and outcomes of patients with patient who undergo TAVR with chronic total occlusion vs. no chronic total occlusion.

Variable	No CTO (*n* = 1,320)	CTO (*n* = 167)	*p*-value
Median procedure time	27 (22–36)	27 (22–37)	0.83
Complication	271 (20.5%)	31 (18.6%)	0.55
Acute renal failure (requiring dialysis)	4 (0.3%)	2 (1.2%)	0.086
Post operative stroke or transient ischaemic attack	31 (2.4%)	3 (1.8%)	0.65
3° atrioventricular block/PM need	143 (10.8%)	18 (10.8%)	0.98
Aortic dissection	2 (0.2%)	0 (0%)	0.62
Tamponade	24 (1.8%)	0 (0%)	0.079
Circulatory arrest	3 (0.2%)	0 (0%)	0.54
Switch to surgery	3 (0.2%)	0 (0%)	0.54
Valvular dysfunction periintervention	15 (1.1%)	0 (0%)	0.17
Annulus rupture	4 (0.3%)	0 (0%)	0.48
Vascular complication	31 (2.4%)	5 (3.0%)	0.61
Operative death (on table death)	2 (0.2%)	0 (0%)	0.62
Mean EF at discharge	55.2 ± 9.6	50.3 ± 12.3	<0.001
Aortic regurgitation at discharge			0.40
None	685 (71.4%)	75 (77.3%)	
Minimal	167 (17.4%)	16 (16.5%)	
Mild	98 (10.2%)	6 (6.2%)	
Moderate	10 (1.0%)	0 (0%)	
Coronary occlusion	7 (0.5%)	1 (0.6%)	0.91
Median rapid pacing time	20 (12–27)	20 (0–25)	0.27
Median fluoroscopy time (IQR)	5 (4–9)	5 (4–9)	0.23
Median contrast volume (IQR)	93 (74–125)	95 (74–122)	0.79
Death in-hospital	14 (1.1%)	1 (0.6%)	0.58
Death at 30 days	33 (2.5%)	4 (2.4%)	0.94
Death at follow up	85 (6.4%)	10 (6.0%)	0.82

The rates complications did not differ significantly between the two groups (*p* = 0.87 and *p* = 0.55, respectively). However, there was a non-significant trend towards a higher incidence of acute renal failure in the CTO group (1.2%, *n* = 2) compared to the no CTO group (0.3%, *n* = 4) (*p* = 0.086). Post-operative dialysis, post-operative stroke or transient ischemic attack, third-degree atrioventricular block/need for pacemaker implantation, aortic dissection, tamponade, circulatory arrest, switch to surgery, valvular dysfunction periintervention, annulus rupture, vascular complication, and operative death did not differ significantly between the two groups (all *p* > 0.05).

At discharge, patients in the CTO group had a significantly lower mean ejection fraction (50.3% ± 12.3%) compared to the no CTO group (55.2% ± 9.6%) (*p* < 0.001). On the other hand, the presence of aortic regurgitation at discharge did not differ significantly between the groups (*p* = 0.40) and there was no significant difference in the presence of mitral regurgitation at discharge (*p* = 0.31).

**Figure 1:**
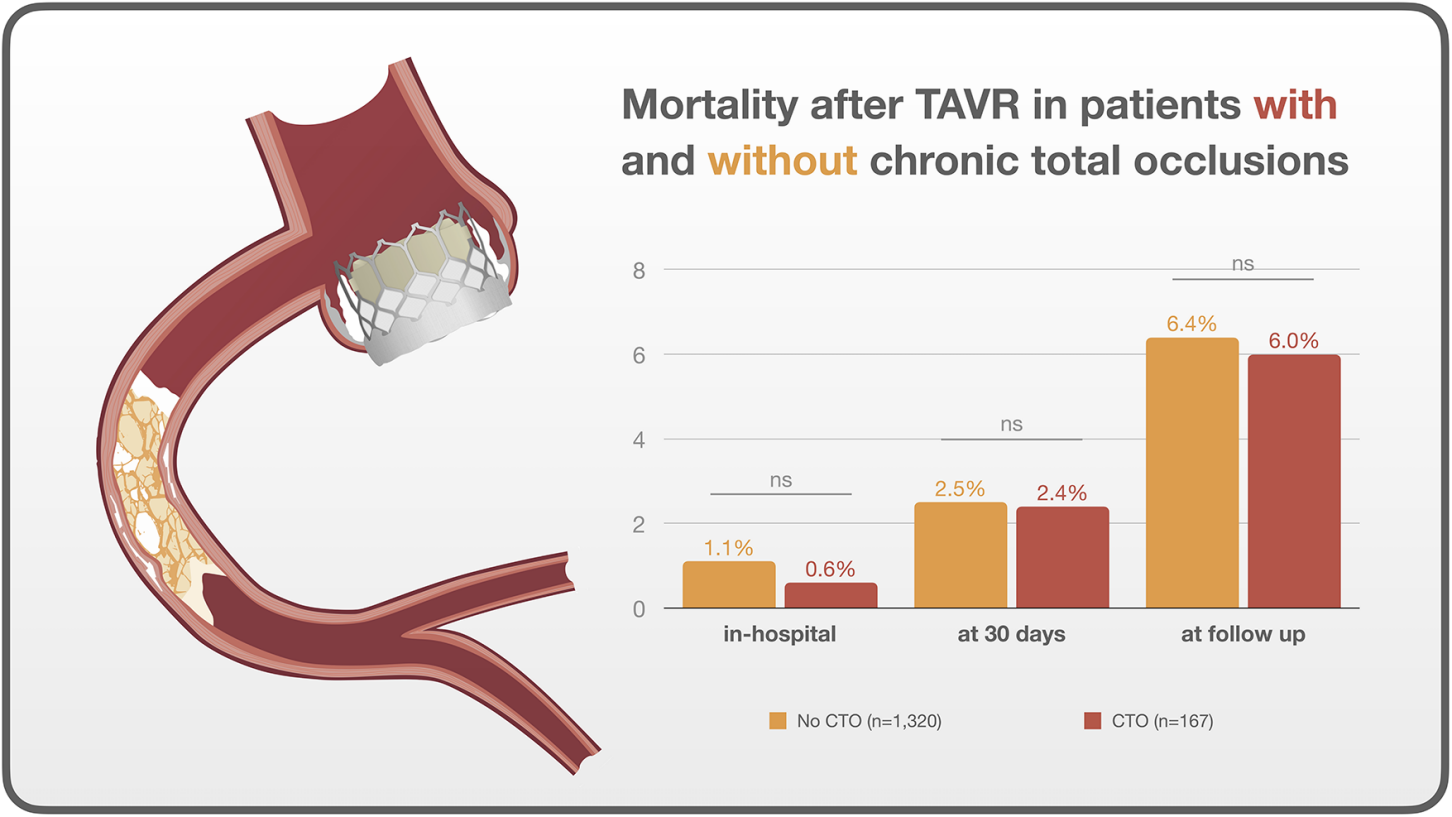
Central illustration. Outcomes after TAVR in patients with and without chronic total occlusions.

**Figure 2:**
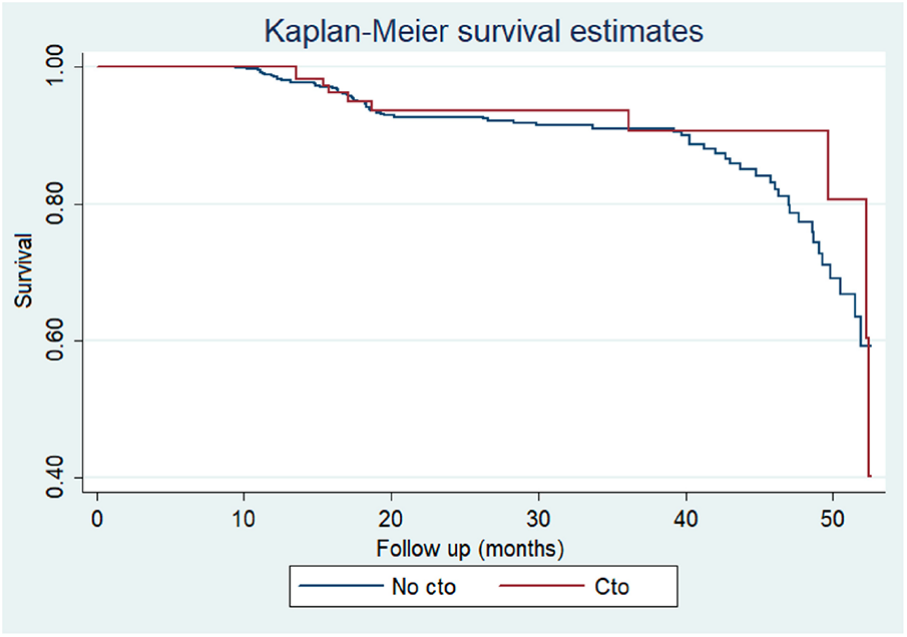
Kaplan–Meier survival curves according to presence of chronic total occlusions in patients receiving TAVR.

Other procedural variables and outcomes, including coronary occlusion, median rapid pacing time, fluoroscopy time, contrast volume, in-hospital death, death at 30 days, and death at median follow-up after 1.9 years, did not show significant differences between the two groups (all *p* > 0.05) (Figures 1, 2).

Table 3 provides in-depth information about CTO lesions in patients who underwent TAVR. In total, 167 patients (11.2% of whole sample) undergoing TAVR had concomitant CTO. Out of these, 91 patients had a single CTO, 43 had two CTOs, and 33 presented with three CTOs (CTO 1, CTO 2 and CTO 3). A majority of these patients (*n* = 124, 74.3%) had their CTO revascularized. Among the revascularized CTO cases, 16 patients underwent PCI while 109 patients underwent CABG.

**Table 3:** Description of chronic total occlusion lesions if patients who undergo TAVR.

Variable	Total patients with CTO (*n* = 167)
Revascularized CTO	124 (74.3%)
Revascularized with PCI	16 (9.6%)
Revascularized with CABG	109 (65.2%)
Patent at time of TAVR	89 (53.3%)
Occluded at time of TAVR	20 (12.0%)
Vessel of CTO 1	
Left anterior descending artery	85 (50.9%)
Left circumflex artery	20 (12.0%)
Right coronary artery	52 (31.1%)
Obtuse marginal artery	3 (1.8%)
Posterior left ventricular artery	2 (1.2%)
Posterior descending artery	1 (0.6%)
Right posterior descending artery	0 (0%)
Ramus intermedius	3 (1.8%)
Other	1 (0.6%)
Location of CTO1	
Proximal	112 (67.5%)
Mid	46 (27.7%)
Distal	8 (4.8%)
Other	1 (0.6%)
Viability of CTO1 (*n* = 176)	
Viable	134 (80.2%)
Not viable	26 (15.6%)
Unknown	7 (4.2%)
Vessel of CTO 2	
No CTO	91 (54.5%)
Left anterior descending artery	10 (6.0%)
Left circumflex artery	32 (19.2%)
Right coronary artery	22 (13.2%)
Obtuse marginal artery	6 (3.6%)
Posterior left ventricular artery	1 (0.6%)
Posterior descending artery	0 (0%)
Right posterior descending artery	2 (1.2%)
Ramus intermedius	3 (1.8%)
Location of CTO 2	
No CTO	91 (54.5%)
Proximal	58 (34.9%)
Mid	13 (7.8%)
Distal	5 (3.0%)
Viability of CTO 2 (*n* = 76)	
Viable	61 (80.3%)
Not viable	13 (17.1%)
Unknown	2 (2.6%)
Vessel CTO 3	
No CTO	134 (80.2%)
Left anterior descending artery	3 (1.8%)
Left circumflex artery	4 (2.4%)
Right coronary artery	18 (10.8%)
Obtuse marginal artery	2 (1.2%)
Posterior left ventricular artery	0 (0%)
Posterior descending artery	1 (0.6%)
Right posterior descending artery	2 (1.2%)
Ramus intermedius	3 (1.8%)
Location CTO 3	
No CTO	134 (80.2%)
Proximal	24 (1.6%)
Mid	4 (0.3%)
Distal	5 (0.3%)
Viability of CTO 3 (*n* = 76)	
Viable	24 (14.4%)
Not viable	9 (5.4%)
Unknown	0 (0%)
Dominance	
Left dominant	35 (21.0%)
Right dominant	53 (31.7%)
Co-dominant	78 (46.7%)
Unknown	1 (0.6%)
Donor vessel 1 to CTO	
No donor vessel	105 (62.9%)
Left anterior descending artery	29 (17.4%)
Left circumflex artery	13 (7.8%)
Right coronary artery	4 (2.4%)
Right diagonals	3 (1.8%)
Other	1 (0.6%)
Donor vessel 2 to CTO	
No donor vessel	130 (77.8%)
Left anterior descending artery	14 (8.4%)
Left circumflex artery	15 (9.0%)
Right coronary artery	6 (3.6%)
Right diagonals	1 (0.6%)
Right marginals	1 (0.6%)
Other diseased vessel 1	
None	84 (50.3%)
Left anterior descending artery	23 (13.8%)
Left circumflex artery	10 (6.0%)
Right coronary artery	22 (13.2%)
Obtuse marginal	5 (3.0%)
Right diagonals	4 (2.4%)
Left main stem	11 (6.6%)
Ramus intermedius	7 (4.2%)
Unknown	1 (0.6%)
Location of other diseased vessel 1	
None	87 (52.1%)
Proximal	55 (32.9%)
Mid	12 (7.2%)
Distal	12 (7.2%)
Unknown	1 (0.6%)
Other diseased vessel 2	
None	136 (81.4%)
Left anterior descending artery	4 (2.4%)
Left circumflex artery	6 (3.6%)
Right coronary artery	8 (4.8%)
Obtuse marginal	4 (2.4%)
Right diagonals	3 (1.8%)
Left main stem	3 (1.8%)
Ramus intermedius	1 (0.6%)
Unknown	2 (1.8%)
Location of other diseased vessel 2	
None	135 (80.8%)
Proximal	24 (14.4%)
Mid	2 (1.2%)
Distal	3 (1.8%)
Unknown	3 (1.8%)

Regarding the CTO characteristics, 65.2% (*n* = 109) had an occluded CTO at the time of TAVR, while 53.3% (*n* = 89) had a revascularized CTO. The most common vessel involved in the CTO was the left anterior descending artery (50.9%, *n* = 85), followed by the right coronary artery (31.1%, *n* = 52) and the left circumflex artery (12.0%, *n* = 20). The location of the CTO was predominantly proximal (67.5%, *n* = 112) or mid (27.7%, *n* = 46), and the viability of the CTO was confirmed in 80.2% of cases. For patients with multiple CTOs, the respective vessels involved and the location, and viability of the CTOs are described in the Table 3. It also provides additional information on the donor vessels supplying blood to the CTO, other diseased vessels, and their respective locations.

In Table 4, we present a subgroup analysis evaluating the impact of CTO revascularization and viability on various outcomes in the study population. The table includes different groups and compares their respective outcomes, as well as the corresponding *p*-values for statistical significance.

**Table 4:** Subgroup analysis evaluating the impact of chronic total occlusion revascularization and viability.

Group	Operative mortality	Mortality at discharge	30-day mortality	Follow up mortality	Post-op stroke	Perioperative MI	Vascular complication	Major bleed
No CTO (*n* = 1,320) vs. revasc CTO vs. unrevasc CTO								
No CTO (*n* = 1,320)	2 (0.2%)	14 (1.1%)	33 (2.5%)	85 (6.4%)	25 (1.9%)	0 (0%)	4 (0.3%)	34 (2.6%)
Revasc CTO (*n* = 43)	0 (0%)	0 (0%)	1 (2.3%)	3 (7.0%)	1 (2.3%)	0 (0%)	2 (4.7%)	2 (4.7%)
Unrevasc CTO (*n* = 124)	0 (0%)	1 (0.8%)	3 (2.4%)	7 (5.7%)	2 (1.6%)	0 (0%)	2 (1.6%)	2 (1.6%)
*p*-value	0.88	0.77	0.99	0.93	0.95	–	<0.001	0.55
CTO with viability vs. CTO no viability								
CTO with viability (*n* = 104)	0 (0%)	1 (1.0%)	1 (1.0%)	4 (3.9%)	2 (1.9%)	0 (0%)	2 (1.9%)	2 (1.9%)
CTO no viability (*n* = 17)	0 (0%)	0 (0%)	2 (11.8%)	3 (17.7%)	0 (0%)	0 (0%)	0 (0%)	0 (0%)
*p*-value	–	0.7	0.008	0.024	0.56	–	0.56	0.56
CTO revasc prox LAD vs. CTO no revasc prox LAD								
CTO revasc prox LAD (*n* = 77)	0 (0%)	0 (0%)	0 (0%)	1 (12.5%)	0 (0%)	0 (0%)	0 (0%)	0 (0%)
CTO no revasc prox LAD (*n* = 8)	0 (0%)	0 (0%)	1 (1.3%)	4 (5.2%)	1 (1.3%)	0 (0%)	2 (2.6%)	1 (1.3%)
*p*-value	–	–	0.75	0.4	0.75	–	0.65	0.75

CTO, chronic total occlusion; MI, myocardial infarction; LAD, left anterior descending artery.

The first comparison involved three groups: “No CTO” (*n* = 1,320), non-revascularized CTO “Non-revasc CTO” (*n* = 43), and revascularized CTO “Revasc CTO” (*n* = 124). These groups were evaluated based on multiple outcome measures, including mortality rates, post-op stroke, myocardial infarction, vascular complications, and major bleeding. Eventually, no statistically significant differences were found among the groups for any of the outcomes. In the next analysis we combined the “No CTO” and “Revasc CTO” groups, creating the “No CTO + Revasc CTO” group (*n* = 1,444), and compared it to the “Unrevasc CTO” group (*n* = 43). Again, no statistically significant differences were observed between the groups.

Another comparison was made between patients with CTO, differentiating them based on the presence of viability. The analysis revealed that patients without viability (*n* = 17) exhibited higher mortality rates at both 30-day and follow-up periods (*p* = 0.008 and *p* = 0.024, respectively). Additionally, two groups were examined: “No CTO + CTO revasc with viability” (*n* = 1,441) and “CTO unrevasc + CTO no viability” (*n* = 46). Once again, no statistically significant differences were found between these groups for any of the outcome measures, demonstrating that different approaches did not significantly impact the overall outcomes.

## Discussion

Our analysis presents several key findings. Firstly, patients with coronary CTOs undergoing TAVR represent a different patient population than those without.

Of note, similarly to previously published results (26, 27) relevant comorbidities were significantly more prevalent in the CTO vs. no CTO cohort encompassing arterial hypertension (85.6% vs. 78.8%), dyslipidemia (88% vs. 65.8%), diabetes mellitus (40.1% vs. 32.7%). Fittingly, median creatinine levels were slightly albeit significantly higher in the CTO compared to no CTO cohort (1.2 vs. 1.1 mg/dl). Furthermore, presence of angina was significantly more frequent in the CTO group (8.4% vs. 3.4%) and a higher proportion of patients with prior myocardial infarction (10.8% vs. 2.3%) was detected in that group. Consistently, PCI and CABG were significantly more performed in the CTO group (47.9% and 65.9%) compared to the no CTO group (35.6% and 1.8%), respectively. Notably, patients with CTO, compared to those without CTO, exhibited a significantly higher prevalence of left main stem disease (57.5% vs. 4.1%) and significantly higher perioperative EuroSCORE I and EuroSCORE II mortality scores.

Furthermore, the mean aortic valve gradient was significantly higher in the no CTO vs. CTO group (45.8 vs. 36.8 mmHg). However, there was no statistically significant difference in the aortic valve area between the two groups, with a same median value of 0.7 cm^2^ both the CTO and no CTO. These findings correspond with earlier outcomes derived from the sole study pertaining to this subject conducted by Howard and coworkers (27). Interestingly, patients in the CTO group had a significantly lower mean LVEF at discharge compared to the no CTO group (50.3% vs. 55.2%). This observation is consistent with previous research findings and may partially explain the lower mean aortic valve gradients in this group (27). The underlying reasons for this difference in contractile function are unclear, however, it could be attributed to a likely compromised myocardial function in the presence of CTO or a greater prevalence of related conditions in the CTO cohort, such as prior myocardial infarction and need for surgical or percutaneous revascularization. This finding is in agreement with several observational studies affirming the adverse prognostic impact of CTO on LVEF within other populations (31).

Secondly, our results suggest that the concomitant presence of a CTO in a population of patients with severe AS undergoing TAVR was not associated with adverse clinical outcomes. This effect was consistent although some baseline characteristics and risk stratification scores might suggest worse prognosis of the patients with CTO compared to their counterparts. Of importance, our additional analysis showed that the prognosis was unaffected even if the CTO was revascularized. However, we observed a trend towards a higher incidence of acute renal failure in the CTO group (1.2%) compared to the no CTO group (0.3%) which did not reach statistical significance.

Thirdly, this analysis compared patients with CTOs undergoing TAVR based on the presence or absence of myocardial viability. Our subanalysis revealed significantly higher 30-day and follow-up mortality rates in the patients with CTO and no myocardial viability compared to those with viable myocardium while no significant differences were observed for other outcomes.

Several studies in population of patients with CAD have highlighted the impact of CTOs on patient outcomes (20–24, 31). It was postulated that patients with CTOs face an increased risk of fatal future cardiac events due to the “*double jeopardy”* phenomenon, wherein acute occlusion of a donor vessel supplying collateral blood flow to a region beyond the CTO poses a threat to a larger area of myocardium (23). This is substantiated by the three-fold increase in 30-day mortality among STEMI patients treated with concomitant CTO in a non-culprit artery (23). The mortality significantly increases to a high 52% when there is acute closure in the collateral donor artery. However, this concept was recently challenged by investigations led by Scholz and coworkers (32).

Malignant arrhythmias also seem to contribute significantly to cardiovascular death in patients with CTOs (22, 33, 34). Additionally, non-revascularized CTOs may be associated with impaired left ventricular ejection fraction (LVEF), a well-established prognostic marker for major adverse cardiovascular events (18). However, it is important to note that patients undergoing TAVR have different myocardial characteristics compared to those with ischemic heart disease, as severe aortic stenosis patients often exhibit left ventricular hypertrophy (LVH) and abnormalities in the coronary microcirculation. LVH predisposes to ischemia and promotes the development of coronary collateral circulation, which exerts a protective effect on patient outcomes regardless of disease burden, even in those with extensive ischemic heart disease (35).

Despite all the mentioned considerations and postulated mechanisms, our study did not find associations of concomitant CTO with adverse outcome in patients with severe AS undergoing TAVR.

However, it is important to consider that many patients that undergo TAVR are octogenarians and naturally won't benefit from CTO revascularization in terms of mortality, where a potential prognostic benefit might be present after several years (24). Therefore, it is imperative to exercise caution when interpreting the findings of this study in relation to younger patients undergoing TAVR. Importantly, this group of patients are projected to expand significantly in the coming years due to advancements in techniques and emerging indications. Another contributing factor to these results could be attributed to the relatively large size of the revascularized CTO group, juxtaposed with the comparatively modest size of the non-revascularized CTO group. Hence, outcomes of lesser occurrence, such as mortality might be underpowered to detect a significant difference. In the event of a subtle effect size a rather small study like ours might fail to discern it. Therefore, a prospective study with larger sample size becomes imperative to address this significant inquiry.

Currently, there is a recommendation for revascularization in patients in TAVR patients with significant CAD, however, there exists insufficient evidence on the significance and optimal timing of percutaneous coronary intervention in these individuals due to conflicting study findings (16, 17, 36). In theory, revascularization of a CTO b before TAVR has the potential to prevent periprocedural myocardial infarction and hemodynamic deterioration during the rapid pacing period. However, it is important to consider that there are significant risks associated with CTO-PCI before TAVR and patients may experience serious complications. Recently, Rheude et al. (37) compared PCI timing strategies in a prospective TAVR registry and showed a survival benefit of revascularization after the valve procedure. Some postulated causal mechanisms may apply to CTO-PCI as well. Notably, intermittent hemodynamic compromise during PCI procedures due to severe AS was associated with cerebral ischemia leading to higher stroke rates when performing PCI before TAVR. Due to the complex nature and longer durations with CTO-PCI, this fact could be particularly relevant in this setting.

To the best of our knowledge, the present analysis is the first study providing detailed information on revascularization status and viability of the myocardium in patients with coronary CTOs undergoing TAVR. Nonetheless, exploratory subgroup analyses failed to show an effect of revascularization even in the setting of viable myocardium and in proximal LAD-lesions compared to others. However, due to the small numbers in the investigated cohorts, these analyses are spurious and only hypotheses generating. Therefore, future investigation should be directed towards analyzing the potential impact of revascularization, myocardial viability, and territory involved in patients with CTO undergoing TAVR (27, 30).

Deciding whether to proceed with CTO-PCI and TAVR is a complex task that requires the involvement of a multidisciplinary team specializing in heart conditions and individualized decision making. It would be beneficial for more extensive studies, preferably randomized controlled trials, to be conducted to gain deeper insights into this matter. RCTs are necessary since randomization assumes significance as it helps eliminate any potential biases resulting from selective factors affecting decision-making in this context.

### Limitations

We are aware that our study has significant limitations. First, this is a retrospective single center experience. Consequently, all limitations and unfavorable aspects inherent in such a setup also exert an influence on our research findings. Second, even though our study provides detailed information regarding CTO vessel characteristics, viability of territory and revascularization status, it is important to note, that the total sample size was relatively limited. Furthermore, even though the primary indication for CTO revascularization in CCS is symptom control our data lacks information on the severity of angina.

The majority of the study patients with CTOs also had severe concomitant CAD, including left main disease. This may introduce a potential bias in the findings, particularly due to the lack of detailed information available on the management of concurrent lesions.

## Conclusions

In conclusion, this analysis suggests concomitant CTO lesions *per se* were not associated with increased mortality in patients receiving TAVR. However, TAVR patients with CTO and viable myocardium had better 30-day and long-term mortality outcomes compared to patients with CTO and no viability. Future studies conducted in larger cohorts and RCTs are warranted to confirm our results.

## Data Availability

The raw data supporting the conclusions of this article will be made available by the authors, without undue reservation.
